# High recurrence of lymphedema and influencing factors in discharged breast cancer patients during the COVID‐19 pandemic: A multicenter, cross‐sectional survey

**DOI:** 10.1002/cam4.4737

**Published:** 2022-05-11

**Authors:** Xin Peng, Renwang Chen, Zhenli Rao, Yi Yang, Yunli Yan, Ying Xia, Ting Wang, Jianying Wang, Fang Lu, Hong Pan, Yan Liu, Jing Cheng, Qin He

**Affiliations:** ^1^ Cancer Center, Union Hospital, Tongji Medical College Huazhong University of Science and Technology Wuhan China; ^2^ Breast Center Hubei Cancer Hospital Wuhan China; ^3^ Thyroid Breast Surgery, Tongji Hospital, Tongji Medical College Huazhong University of Science and Technology Wuhan China; ^4^ Department of Nursing Wuhan First Hospital Wuhan China; ^5^ Thyroid Breast Surgery Renmin Hospital of Wuhan University Wuhan China; ^6^ Department of Nursing Zhongnan Hospital of Wuhan University Wuhan China; ^7^ Thyroid Breast Surgery Wuhan Central Hospital Wuhan China; ^8^ Department of Nursing Wuhan Third Hospital Wuhan China; ^9^ Public Health Section, Union Hospital, Tongji Medical College Huazhong University of Science and Technology Wuhan China

**Keywords:** breast cancer, COVID‐19, lymphedema, norman Questionnaire, PROs

## Abstract

**Background:**

There has been a delay in the detection and treatment of lymphedema in breast cancer patients during the lockdown owing to quarantine and limited social activity. Moreover, this scenario has caused psychosocial issues in these patients. Given that there is scarce information on the prevalence and influence of lymphedema during the coronavirus disease (COVID‐19) pandemic, we aimed to estimate the prevalence of lymphedema recurrence and its influencing factors among discharged breast cancer patients during the COVID‐19 pandemic.

**Methods:**

This was a multicenter, cross‐sectional, hospital‐based survey of discharged breast cancer patients was conducted during the COVID‐19 pandemic in eight first‐class hospitals in Wuhan, China. The Norman Questionnaire was used to assess lymphedema. Univariable and multivariable binary logistic regression analyses were performed to identify factors influencing moderate or severe lymphedema. Differences in living characteristics, anxiety, and depression were compared between the no/mild lymphedema group and the moderate/severe lymphedema groups. Preferences for lymphedema management during the pandemic were determined.

**Results:**

Overall, 202 patients were included in this study, and 191 of them reported recurrent lymphedema (prevalence: 94.6%, 95% confidence interval [CI] 90.5% to 97.3%). Among them, 134 and 57 had mild and moderate/severe lymphedema, respectively. In 191 patients, the main symptoms were swelling (140; 69.3%) and pain (56, 27.7%). Multivariable regression showed that older age (odds ratio [OR], 1.06; 95% CI: 1.02–1.10), radical surgery (OR = 4.35, 95% CI: 1.54–12.50), and fully complete radiotherapy (OR = 2.62, 95% CI: 1.17–5.87, *p* = 0.019) were associated with an elevated risk of moderate/severe lymphedema. The moderate/severe lymphedema group experienced a higher rate of anxiety and depression than the no/mild lymphedema group did. Patients equally preferred treatment in the hospital and self‐care at home.

**Conclusion:**

During the COVID‐19 pandemic, high prevalence of lymphedema was observed in patients Age, radical surgery and fully completed radiotherapy were associated with increased risk of severer lymphedema. Meanwhile, the patients with severe lymphedema experienced psychological distress. While the Covid‐19 pandemic was still raging, continuous efforts should be made to identify patient at risk of lymphedema and distribute feasible guidance and education for self‐management in lymphedema.

## 1 BACKGROUND

In December 2019, the first case of coronavirus disease (COVID‐19) was identified in Wuhan City, which was the epicenter of the COVID‐19 outbreak in China. COVID‐19 spread rapidly during a short period and has been recognized as a global pandemic by the World Health Organization.^1^, ^2^ At the beginning of the outbreak, there were limited medical resources; however, a large number of patients were infected with COVID‐19. Approximately 51 local hospitals in Wuhan City were transformed into COVID‐19‐designated hospitals for the treatment of patients affected with COVID‐19. Patients with other diseases had a delay in receiving adequate and timely treatment.^3^, ^4^ Patients with breast cancer are an important group of patients worthy of special attention.

According to global cancer statistics 2020, breast cancer has become the most prevalent cancer in women worldwide.^5^ Breast tumors tend to spread lymphatically and hematologically, and the axillary lymph nodes are usually affected first. After surgery, some or all of the axillary lymph nodes under the arm might be dissected. Meanwhile, possible radiation treatment to regional lymph nodes may cause scarring and blockages in the underarm. The interruption of the axillary lymphatic channel puts patients at a high risk of developing breast cancer‐related lymphedema (BCRL). A systematic review of 19 studies that assessed 3035 breast cancer patients showed that 23.43% of the patients developed BCRL.^6^ The type of axillary lymph node dissection influences the number of removed nodes and is a key factor for determining the risk.^7^ After surgery, patients might develop subclinical edema within a few months, which is a factor influencing disease progression to lymphedema.^8^ A network‐based meta‐analysis demonstrated that the addition of radiation therapy to regional nodes could increase the risk of lymphedema.^9^ Although taxane‐based chemotherapy may induce fluid retention, inconsistent evidence suggests that chemotherapy is an influencing factor.^8^, ^10^ Additionally, a high body mass index (BMI) was significantly correlated with increased rates of lymphedema.^11^

Without timely treatment, the swelling would induce skin sores, infection in the affected area, or other problems.^12^ The recurrence of BCRL could further worsen its impacts.^13^ Early identification and treatment of lymphedema are critical to prevent poor prognosis. However, during the COVID‐19 pandemic, patients with breast cancer in Wuhan City experienced a long lockdown and a passive delay in the diagnosis and treatment of lymphedema. Therefore, it is of great significance to survey lymphedema status among patients with breast cancer who are treated for lymphedema. During the COVID‐19 pandemic, patients may be quarantined at home, with limited social activity. A high prevalence of depression and anxiety has been observed in the general population.^14^ A literature review showed that BCRL increases psychosocial issues.^15^ Therefore, it is also essential to investigate the impact of lymphedema on patients’ quality of life. However, to the best of our knowledge, only a limited number of studies have examined the prevalence and influence of lymphedema during the COVID‐19 pandemic.

Self‐reported symptoms are considered suitable for early detection of BCRL.^13^ In this study, we used the validated Norman Lymphedema Questionnaire^16^ and performed a multicenter cross‐sectional survey in eight first‐class hospitals in Wuhan City with an aim to investigate the prevalence of lymphedema and its influencing factors in patients with breast cancer treated for lymphedema.

## 2 MATERIALS AND METHODS

### 2.1 Study design, setting, and participants

Between May 27, 2020, and June 10, 2020, a multicenter, cross‐sectional, hospital‐based survey was conducted in eight first‐class hospitals in Wuhan, China. This study was reviewed and approved by the Ethical Review of Tongji Medical College of Huazhong University of Science and Technology. A convenience sampling method was adopted to select patients with breast cancer. Patients were eligible for inclusion if they met the following criteria: (1) female patients who were diagnosed with breast cancer before COVID‐19; (2) patients who were discharged after treatment; (3) patients who had lymphedema before the COVID‐19 pandemic and the lymphedema had been effectively treated and the affected limbs had recovered to normal before January 2020; (4) patients in whom lymphedema‐related intervention was interrupted during COVID‐19 from January to April 2020; and (5) patients who agreed to provide informed consent and volunteered to participate in this study. The exclusion criteria were (1) infection with COVID‐19 and (2) new recurrence or metastasis of breast cancer.

### 2.2 Data collection

A self‐administered electronic questionnaire was designed to collect the data. Structural and unified instructions were prepared to explain the purpose, meaning, and method of filling the questionnaire. The link embedded in the QR code was distributed through WeChat (a famous online communication tool in China). One WeChat account could complete the questionnaire only once, and the time consumed was recorded for further validation.

### 2.3 Baseline characteristics

Demographic characteristics included age, height, weight, surgery (yes/no), type of surgery (radical surgery/others), radiotherapy (never/fully completed/interrupted), and use of taxane drugs or hormones in the past 6 months(yes/no), all of which were considered potential influencing factors.

### 2.4 Measures of lymphedema and its symptoms

The previously validated Norman Lymphedema Questionnaire was used to detect lymphedema symptoms in the participants.^16^ Swelling status in the past 3 months was assessed in three parts (hand, forearm, and upper arm). For each part, the scores ranged from 0 to 3. A score of 0 indicated no swelling, 1 indicated a slight swelling that was perceived only by the patient, 2 indicated moderate swelling that was noticed by the patient’s caregivers during daily life, and 3 indicated a serious swelling that was noticed by strangers. The total score ranges from 0 to 9. According to the total score, lymphedema status was classified as no lymphedema (score 0), mild lymphedema (scores 1–4), and moderate‐to‐severe lymphedema (scores 5–9). Patient‐reported outcomes for breast cancer were summarized, and items about lymphedema symptoms were extracted from a review.^17^ Seven symptoms were included for assessment: swelling, pain, heaviness, numbness, stiffness, movement restriction, and asymptomatic.

### 2.5 Influence of BCRL recurrence on daily life and treatment preference

To survey the influence of lymphedema on the daily life and treatment preference in breast cancer patients during the COVID‐19 pandemic, we asked participants to evaluate the current status of BCRL on changes in manual labor (no change/increase/decrease), use of protective measures for the affected limbs (multiple choice question: none/keep skin clean and intact/no lift of heavy objects/postoperative functional exercise), and living conditions (multiple choice question: no exercise at home/excessive housework/accidental injuries/skin allergies/fungal skin infections of the hands). The Hospital Anxiety and Depression Scale (HADS) was used to assess anxiety and depression in the general population.^18^ It contains 14 items (seven for anxiety and seven for depression). A cutoff score of greater than eight suggested the presence of anxiety and depression, which required medical attention. Meanwhile, the participants’ preferred method for lymphedema treatment in the post‐epidemic era was inquired, including on‐site service, community service, hospital treatment, online consultation, self‐care at home, and family’s help.

### 2.6 Statistical analysis

Two previous systematic reviews and meta‐analyses showed that approximately one in every five patients developed BCRL following breast cancer treatment.^6^, ^19^ We assumed the prevalence of BCRL to be 25%, and the corresponding precision was set to half of the prevalence. To produce a two‐sided 95% confidence interval with a width equal to 0.125 when the sample proportion was 0.25, 182 patients were required, with a risk of 5% type I error. A validity rate of 95% was assumed for the collected questionnaires; the final sample size was 195 patients.

The data were extracted from an online questionnaire. Statistical analysis was performed using the Statistical Package for the Social Sciences software (version 22.0; SPSS). First, we performed statistical description and group comparisons. Continuous variables were described using means and standard deviations, and categorical variables were described using frequencies (percentages). Participants were classified into two groups (none/mild lymphedema and moderate/severe lymphedema). Univariate and multivariate binary logistic regression analyses were conducted to explore the potential and independent factors for moderate‐to‐severe lymphedema. Odds ratios (ORs) and the corresponding 95% confidence intervals (95% CIs) are presented.

The influence of lymphedema severity on patients’ daily life, including changes in manual labor, protective measures for the affected limbs (multiple choice questions), living conditions (multiple choice questions), and anxiety and depression, was further investigated, and the differences between the two groups were compared using the chi‐square test or Fisher’s exact test, as appropriate. To correct the family‐wise error rate, Bonferroni correction was applied for four items in the protective measures, five items in the living conditions, and two items in the HADS. A two‐sided *p* value of less than 0.05 was considered statistically significant.

## 3 RESULTS

### 3.1 Patient characteristics

A total of 228 questionnaires were collected, and 16 participants were excluded for taking time of less than 90 s. A total of 202 records were included in the final analysis. The included patients had a mean age of 51.34 years old and a mean BMI of 23.43 kg/m^2^. Moreover, 145 (71.8%) patients underwent radical surgery. Affected by the COVID‐19 pandemic, only 121 (59.9%) patients underwent fully complete radiotherapy, 71 (35.1%) patients did not receive radiotherapy, and 10 (5.0%) patients underwent interrupted radiotherapy. Fifty‐five (22.3%) patients had used taxane or hormone therapy in the past 6 months (Table 1).

**TABLE 1:** Demographic characteristic of discharged breast cancer patients during COVID‐19

Variable	Description
Age, years, mean ± SD	51.34 ± 10.07
BMI, kg/m^2^, mean ± SD	23.43 ± 3.08
Surgery until now (months)	29.76 ± 27.67
Type of surgery, *n* (%)	
Radical surgery	145 (71.8%)
Others	57 (28.2%)
Radiotherapy situation, *n* (%)	
Never	71 (35.1%)
Fully completed	121 (59.9%)
Interrupted	10 (5.0%)
Medication, *n* (%)	
Used	45 (22.3%)
Never used	121 (59.9%)
Unknown	36 (17.8%)

*Note*: Medication, the use of taxane drugs or hormones in the past 6 months.

Abbreviations: BMI, body mass index; COVID‐19, Coronavirus Disease 2019.

### 3.2 Prevalence of lymphedema and self‐reported symptoms

Of the 202 participants, 191 reported recurrent lymphedema, including 134 mild participants and 57 moderate‐to‐severe participants. The prevalence of lymphedema was 94.6% (95% CI 90.5% to 97.3%). Among the 191 patients, the main symptoms were swelling (140, 69.3%), followed by pain (56, 27.7%), heaviness (55, 27.2%), numbness (55, 27.2%), stiffness (31, 15.3%), and movement restriction (31, 15.3%).

### 3.3 Characteristics associated with the degree of lymphedema

Participants were classified into two groups: no or mild lymphedema group (134, 66.3%) and moderate‐to‐severe lymphedema group (57, 28.2%). The descriptions of the two groups are presented in Table 2. Patients’ age was 49.79 ± 10.14 years in the no or mild group and 55.30 ± 8.80 in the moderate‐to‐severe group. Before and after adjustment, older age was significantly associated with more severe lymphedema (crude OR = 1.06 [95% CI: 1.03 to 1.10], *p* = 0.001; adjusted OR = 1.06 [95% CI: 1.02 to 1.10], *p* = 0.004). Among 91.2% patients in the moderate‐to‐severe group, 91.2% underwent radical surgery, and the rate was 61.1% in the other groups. Both univariable and multivariable regression showed that radical surgery methods increased the risk of severe lymphedema using other surgeries as a reference (crude OR = 5.58 [95% CI: 2.10 to 14.82], *p* < 0.001; adjusted OR = 4.35 [95% CI: 1.54 to 12.50], *p* = 0.006). Using no radiotherapy as a reference, fully complete radiotherapy increased the risk (crude OR = 2.16 [95% CI: 1.08 to 4.33], *p* = 0.032; adjusted OR = 2.62 [95% CI: 1.17 to 5.87], *p* = 0.019); however, interrupted radiotherapy did not increase the risk before and after adjustment. In the univariate analysis, increased BMI, duration after surgery, and no use of taxane or hormone therapy were associated with an increased risk of severe lymphedema. They became non‐significant after adjustment.

**TABLE 2:** Logistic regression analysis on the relationship between risk factors and risk of moderate or severe lymphedema

	Degree of lymphedema		Crude OR (95%CI)	*p*	Adjusted OR (95%CI)	*p*
	None or mild (*n* = 145)	Moderate or severe (*n* = 57)				
Age	49.79 ± 10.14	55.30 ± 8.80	1.06 (1.03, 1.10)	0.001	1.06 (1.02–1.10)	0.004
BMI	23.01 ± 2.99	24.33 ± 3.15	1.14 (1.03, 1.27)	0.01	1.08 (0.97–1.21)	0.176
Surgery until now (months)	25.34 ± 24.09	41.15 ± 32.51	1.02 (1.01, 1.03)	<0.001	1.01 (0.99–1.02)	0.155
Surgical method						
Radical surgery	93 (61.1%)	52 (91.2%)	5.58 (2.10, 14.82)	<0.001	4.35 (1.54–12.50)	0.006
Others	52 (35.9%)	5 (8.8%)	1.00		1.00	
Radiotherapy						
Never	57 (59.7%)	14 (24.6%)	1.00		1.00	
Fully completed	79 (54.5%)	42 (73.7%)	2.16 (1.08, 4.33)	0.032	2.62 (1.17–5.87)	0.019
Interrupted	9 (6.2%)	1 (1.8%)	0.45 (0.05, 3.87)	0.468	0.60 (0.05–6.91)	0.685
Medication						
Used	38 (26.2%)	7 (12.3%)	1.00		1.00	
Never used	81 (55.9%)	40 (70.2%)	2.68 (1.10. 6.53)	0.030	1.46 (0.53–3.97)	0.461
Unknown	26 (17.9%)	10 (17.5%)	2.09 (0.70, 6.19)	0.184	1.84 (0.51–6.68)	0.353

*Note*: Medication, the use of taxane drugs or hormones in the past 6 months.

Abbreviation: BMI, body mass index.

### 3.4 Influence of lymphedema on lifestyle and psychological state

During the COVID‐19 pandemic, after lymphedema recurred, less than 20% of patients experienced increased physical labor, and more than 80% took protective measures for the affected limbs. Meanwhile, approximately half of the patients exercised less frequently at home, and nearly one in five patients reported excessive housework during quarantine at home. The rates of accidental injuries, skin allergies, and fungal skin infections of the hands were low. No significant differences in lifestyle were observed between the two groups. After Bonferroni correction, a higher proportion of both anxiety and depression was observed in the moderate‐to‐severe group than in the no or mild group (78.9% vs. 54.4%, 24.6% vs. 9.7%, respectively; Table 3).

**TABLE 3:** Difference in lifestyles and psychological states between no or mild group and moderate or severe group

Parameters	Degree of lymphedema		*p*	*p* ^a^
	None or mild *n* (%)	Moderate or severe *n* (%)		
Manual labor			0.870	0.870
No change	78 (53.8%)	29 (50.9)		
Increase	26 (17.9%)	12 (21.1)		
Decrease	41 (28.3%)	16 (28.1)		
Protective measures for affected limbs				
None	24 (16.6%)	7 (12.3)	0.448	1.000
Keep skin clean and intact	87 (60.0%)	32 (56.1)	0.616	1.000
No lift of heavy objects	114 (78.6%)	50 (87.7)	0.136	0.544
Postoperative functional exercise	64 (44.1%)	21 (36.1)	0.345	1.000
Living conditions				
Less exercise at home	75 (51.7%)	36 (63.2%)	0.142	0.710
Excessive housework	24 (16.6%)	10 (17.5%)	0.865	1.000
Accidental injuries	5 (3.4%)	3 (5.3%)	0.846	1.000
Skin allergies	15(10.3%)	2(3.5%)	0.196	0.980
Fungal skin infections of the hands	3(2.1%)	0(0.0%)	0.654	1.000
Psychological state				
Anxiety	79(54.4%)	45(78.9%)	0.001	0.002
Depression	14(9.7%)	14(24.6%)	0.006	0.012

Abbreviations: BMI, body mass index; COVID‐19, Corona Virus Disease 2019.

^a^ *p* Values were adjusted using Bonferroni correction.

### 3.5 Patients’ needs for lymphedema treatment during the COVID‐19 pandemic

The survey showed that 70.8% of patients hoped to receive treatment at the hospital, even though COVID‐19 greatly limited accessibility. The same rate was observed for need for self‐care at home. Online consultation and family help were secondary options, accounting for approximately 35.1% and 32.7%, respectively. Approximately one in five patients chose community services, and only 11.4% of patients were willing to receive door‐to‐door services.

## 4 DISCUSSION

In this study, the prevalence of recurrent lymphedema during the COVID‐19 pandemic was as high as 94.6%, which was significantly higher than the previously reported incidence.^7^ The participants in this study had experienced BCRL in the past and may have been more likely to develop BCRL again because of persistent risk factors. During the COVID‐19 pandemic, several months of city lockdown and home quarantine were required to prevent the rapid spread of the COVID‐19 virus; however, breast cancer patients with potential BCRL were restricted from visiting the hospital. The long time limit significantly increases the estimated rate. The differential diagnoses of lymphedema are broad. Self‐assessment reported a higher rate of BCRL than formal assessment.^20^ In this study, participants were required to complete the validated Norman Lymphedema Questionnaire online (ranging from 1 to 9). Lymphedema was considered present if the total score was greater than 1. The small cutoff value might also explain the high rate of recurrent BCRL. Lymphedema‐induced swelling, infection, and dysfunction of the limbs seriously affect patients’ quality of life.^21^, ^22^ A national study conducted during the COVID‐19 pandemic showed a higher proportion of infections in cancer patients than the overall incidence of cancer.^23^ An inflammatory process was developed progressively in the limbs affected by lymphedema. The high prevalence of lymphedema during the COVID‐19 pandemic and the possible subsequent consequences should draw our attention to distributing effective and timely interventions to prevent and treat lymphedema.

Unfortunately, the COVID‐19 pandemic is ongoing. Prevention of BCRL is more beneficial than treatment, and identifying potential influencing factors is of great significance. In our study, the risk of severe lymphedema increased by 6% with every additional year of age. The mean age of our sample was approximately 50 years, and decreased physical activity was characterized.^24^ Less frequent exercise would influence the flow of lymph fluid and increase the risk of BRCL. Cancer survivors experience fatigue and barriers to physical activity after treatment.^25^ Home quarantine during the pandemic may further limit patients from exercising. Even though more than half of the patients kept their skin clean and intact and did not lift heavy objects, only approximately two‐fifths of the patients insisted on performing postoperative functional exercise. Nursing staff can provide remote and feasible guidance on functional exercises for older breast cancer patients.

We found that radical mastectomy independently increased the risk of severe lymphedema by approximately four times. It involves the removal of tissue from both breasts, fatty lymphatic tissues, and lymph nodes, which disrupt the lymphatic pathways. The risk of BCRL was associated with the number of nodes removed, and complete axillary lymph node dissection significantly increased the incidence of lymphedema.^26^ Clinicians should carefully determine the extent of axillary surgery and consider potential quarantine. There are various types of surgery for breast cancer, including less invasive sentinel lymph node biopsy (SLNB). A meta‐analysis showed that patients who underwent SLNB had a lower incidence of lymphedema than those who underwent axillary lymph node dissection.^19^ SLNB may be an effective alternative for patients with breast cancer with clinically negative nodes. Fully completed radiotherapy was another significant influencing factor in our study, which is consistent with a large cohort study.^27^ Patients who underwent both lymph node dissection and radiotherapy further presented a higher risk of BCRL than patients who received radiation only.^8^ A large prospective cohort demonstrated that taxane‐based chemotherapy was not associated with an increased risk of lymphedema.^10^ Our study is in agreement with this conclusion. When clinicians and patients make clinical decisions, the treatment effect and risk of BCRL with different treatment methods should be balanced. Patients undergoing radical surgery and/or radiotherapy should be screened prospectively.

In our study, there was no significant difference in manual labor, protective measures, or living conditions between the none/mild and moderate‐to‐severe groups, but patients with moderate‐to‐severe lymphedema experienced a higher proportion of both anxiety and depression. A recent systematic review suggested a considerable prevalence of depression and anxiety among patients with cancer during the COVID‐19 pandemic.^28^ Limited movements of the affected limbs and concerns about delayed treatment during the COVID‐19 pandemic could further explain the high levels of anxiety and depression in this study. BCRL and negative physical symptoms can subsequently result in a decreased quality of life.^29^ Systematic psychological interventions should be conducted in a timely manner to increase psychological resistance. Educational improvement of standard and professional guidance in monitoring swelling and implementing lymphedema drainage can alleviate patients’ anxiety and depression.^30^ Web‐based multimedia interventions can make breast cancer patients receive more information than pamphlet education.^15^ Meanwhile, the preference for private teaching sessions suggests the need for a remote one‐to‐one teaching strategy.^30^ Nonetheless, timely diagnosis and treatment of lymphedema remain effective and direct measures. Our study showed that patients equally preferred treatment at hospital and self‐care at home. Self‐management of breast cancer patients can help them to develop self‐care abilities in the management of lymphedema and psychosocial adaptation.^31^, ^32^ Considering the ongoing pandemic, nursing education for lymphedema should be distributed to patients who will be discharged in the future. Healthcare workers can guide remote rehabilitation and online medical treatment through telemedicine.^33^

This study has several limitations. First, this was a cross‐sectional study, and no causal inference could be made, but it could be considered that influencing factors occurred before recurrent lymphedema, which provided clues for the temporal relationship. Second, data were collected using an online tool, and selection bias might exist because patients who did not use smartphones failed to participate in this study; meanwhile, recall bias and self‐reporting problems might influence precision. Third, details about treatment methods (surgery, radiotherapy, and chemotherapy) were not collected, and we were limited to compare the advantages and disadvantages of different treatment strategies and their corresponding combinations. In addition, changes in quality of life were not estimated using a valid questionnaire; therefore, we could not comprehensively evaluate the influence of lymphedema or make personalized interventions. Finally, the COVID‐19 pandemic is still ongoing, and a longitudinal study is required to identify new problems in breast cancer patients and search for new solutions.

## 5 CONCLUSION

During the COVID‐19 pandemic, a high prevalence of BCRL has been observed in patients with breast cancer. Age, radical surgery, and fully completed radiotherapy were associated with an increased risk of severe BCRL. Patients with severe lymphedema experienced higher levels of psychological distress. The COVID‐19 pandemic is still raging, and continuous efforts should be made to identify patients at high risk of BCRL and distribute feasible guidance and education for self‐management of lymphedema. Our findings can help formulate an optimal nursing plan and treatment for potential lymphedema in breast cancer patients during the ongoing global COVID‐19 pandemic.

## CONFLICT OF INTEREST

The authors declare that they have no competing interest.

## AUTHOR CONTRIBUTIONS

PX developed the idea, designed the study, and provided financial support for the study. PX and CRW designed the questionnaires and drafted the manuscript. RZL, YY, YYL, XY, WT, WJY, LF, PH, and LF were involved in the acquisition of the data. PX and CRW summarized the data and contributed to data interpretation. CJ and HQ critically revised the manuscript for important intellectual content. The corresponding author had full access to all the data in the study and was responsible for submission for publication.

## CONSENT FOR PUBLICATION

Not applicable.

## ETHICAL APPROVAL

This study was approved by Tongji Medical College of the Huazhong University of Science and Technology.

## Data Availability

Availability of data and materials The data being used and analyzed during the current study are available from the corresponding authors upon reasonable request.
